# Use of potentially inappropriate medication and polypharmacy in older adults: a repeated cross-sectional study

**DOI:** 10.1186/s12877-020-1476-5

**Published:** 2020-02-19

**Authors:** Kristine Thorell, Patrik Midlöv, Johan Fastbom, Anders Halling

**Affiliations:** 1 Department of Quality and Development, Karlskrona, Region Blekinge Sweden; 2 Department of Clinical Sciences, Malmö, General Practice/Family Medicine, Lund University, Box 50332, 202 13 Malmö, Sweden; 3 Aging Research Center, Center for Alzheimer Research, Department of Neurobiology, Care Sciences And Society, Karolinska Institute and Stockholm University, Stockholm, Sweden

**Keywords:** Polypharmacy, Potentially inappropriate medications, Number of chronic conditions, Elderly

## Abstract

**Background:**

With age, the number of chronic conditions increases along with the use of medications. For several years, polypharmacy has been found to be on the increase in western societies. Polypharmacy is associated with an increased risk of adverse drug events (ADE). Medications called potentially inappropriate medications (PIM) have also been found to increase the risk of ADEs in an older population. In this study, which we conducted during a national information campaign to reduce PIM, we analysed the prevalence of PIM in an older adult population and in different strata of the variables age, gender, number of chronic conditions and polypharmacy and how that prevalence changed over time.

**Methods:**

This is a registry-based repeated cross-sectional study including two cohorts. Individuals aged 75 or older listed at a primary care centre in Blekinge on the 31st March 2011 (cohort 1, 15,361 individuals) or on the 31st December 2013 (cohort 2, 15,945 individuals) were included in the respective cohorts. Using a chi2 test, the two cohorts were compared on the variables age, gender, number of chronic conditions and polypharmacy. Use of five or more medications at the same time was the definition for polypharmacy.

**Results:**

Use of PIM decreased from 10.60 to 7.04% (*p*-value < 0.001) between 2011 and 2013, while prevalence of five to seven chronic conditions increased from 20.55 to 23.66% (*p*-value < 0.001). Use of PIM decreased in all strata of the variables age, gender number of chronic conditions and polypharmacy. Except for age 80–84 and males, where it increased, prevalence of polypharmacy was stable in all strata of the variables.

**Conclusions:**

Use of potentially inappropriate medications had decreased in all variables between 2011 and 2013; this shows the possibility to reduce PIM with a focused effort.

Polypharmacy does not increase significantly compared to the rest of the population.

## Background

One of the most common forms of treatment, especially in older adults (≥75 years), is medication treatment. The goal of medication therapy is to prevent, treat or cure disease or symptoms of disease. For older adults with multimorbidity and polypharmacy, the effect of medications may not always be positive among this group of patients. Advances in medication development in recent decades have resulted in that the health care system today can prevent, treat and cure more symptoms and diseases than ever before. The developments in medical practice and medication development have significantly contributed to the increase in life expectancy that is seen today [1].

The morbidity burden of the older population increases due to longer life expectancy [2]. With increased multi-morbidity in the population, the use of medications also increases as does the risk of adverse drug events (ADE). Multimorbidity, measured as number of chronic conditions together with number of medications, has been found to be of importance concerning the risk of hospitalisation in an older population [3, 4]. However, very few studies include a morbidity measurement when evaluating quality of drug treatment [5, 6].

Use of multiple medications simultaneously is known as polypharmacy and is commonly defined as the use of five or more medications at the same time. Polypharmacy has been found to be an independent risk factor for adverse drug events and hospitalisations in an older population [7–9]. Adverse drug events from interactions and contraindications can be misinterpreted as new symptoms or diagnoses and generate prescriptions for new medications. This negative spiral of prescribing to treat side effects or interactions is also known as the prescribing cascade and increases the risk of polypharmacy. The risk of a prescribing cascade increases both with interactions (drug-drug) and contraindications (drug-disease) [10, 11]. Nonetheless, polypharmacy is not wrong, per se, as long as the complete medication list is reviewed, and the risk/benefit ratio is considered for the individual patient, which is called appropriate polypharmacy [1, 12].

To increase the non-benefits of medications even more, the population of older adults has a higher sensitivity for medications that are lipophilic or have a high renal elimination compared to a younger and middle-aged population. This is due to physiological changes that occur with age that can lead to altered body fat/water ratio and decreased kidney function. As a result of these changes, the pharmacokinetic and pharmacodynamic properties of medications can be changed and increase the risk of ADE [1, 4].

Medications with a higher risk for ADEs are commonly referred to by the term potentially inappropriate medications (PIM) for older adults. Use of PIM in older adults has been found to lead to increased morbidity and mortality [13, 14]. The definition of PIM for older adults varies between different quality criteria mainly because they are developed in different countries with dissimilar treatment regimens [15]. The two most often used quality criteria are Beers and the Screening Tool of Older Persons’ potentially inappropriate Prescriptions (STOPP) criteria [16, 17]. Many countries have made their own version of quality criteria for older adults based on local therapy traditions. In Sweden, the Swedish National Board of Health and Welfare has published a Swedish version in the report; ‘Quality indicators for good drug therapy in the elderly’. The indicators cover a wide range of different quality issues related to drug treatment in older adults. There are diagnose-related indicators and medication-related indicators [13]. The purpose of the indicators is to facilitate the follow-up of medical treatment in a population of older adults and to evaluate the quality of the treatment. The first medication indicator in the report, ‘Medicines that should be avoided unless there are special reasons’, defines medications that patients, aged 75 years and older, should avoid and are not diagnose related. The criterion includes long-acting benzodiazepines, tramadol, propiomazine and medicines with anticholinergic effect and are defined as PIM in Sweden. The use of PIM should be as low as possible in the population due to the increased risk of ADE [13].

From 2010 to 2014, there was a national information campaign to improve the care of older adults in Sweden [18]. Among many strategies, there was a focus on increasing the knowledge about the risk with use of PIM in older adults aged 75 years and older. The aim of the information campaign aim was to raise awareness around the risk associated with use of PIM among older adults. The campaign was evaluated by measuring use of PIM by defined daily doses in aggregated data [18]. In this study, the effects of the information campaign were analysed in individual based data. The aim was to analyse the prevalence of PIM in an older adult population in different strata of variables of importance for medications use. These variables are: age, gender, number of chronic conditions and number of medications [19, 20]. The study also analysed how that prevalence changed over time during the information campaign.

## Methods

### Setting and study populations

Blekinge is located in the south-eastern corner of Sweden and is one of the smallest counties in the country with approximately 153,000 inhabitants in 2011 and 2013. Almost all inhabitants in Sweden are registered to a primary care centre. The majority of funding for primary health care comes from a specific county council tax, both public (operated by the county council) and private care centres. Both public and private primary care centres were included in the study. We included two cohorts for comparison in this registry based repeated cross-sectional study. For the cohorts, we included individuals aged 75 or older listed at a primary care centre in Blekinge on the 31st March 2011 for the first cohort and, for the second cohort, individuals listed on the 31st December 2013. Cohort 1 comprised 15,361 individuals and cohort 2 comprised 15,945 individuals. The information campaign to improve care of the population of older adults was active between 2010 and 2014. However, due to the possibility of access to data of medication data, the cohorts were chosen for a slightly shorter period. This is because the 31st March 2011 was the earliest date that we had access to a three-month period of medication data within the local register described below. Furthermore, due to changes in how medication data was encrypted in 2014, the 31st December 2013 was chosen to ensure quality of data for the second cohort.

### Data source and measurements

Data on chronic conditions, age and gender in the study were based on anonymised registry information obtained from the County Council of Blekinge from both primary and secondary care.

Use of medications was identified from the county council’s register of dispensed medicines for all inhabitants in Blekinge. Data in this register were received by the County Council from the Swedish eHealth Agency. It contains the same patient level data on prescribed medicines as the National Prescribed Drug Register at the Swedish National Board of Health and Welfare, but the coverage is restricted to the residents in the county [21, 22].

In Sweden, prescribed medicines are prescribed for a maximum period of three months within the high-cost threshold for medicines [23]. Therefore a three-month period was used to construct a medicine list on used medications [24]. If the same drug was dispensed more than once it was still counted only once. The county council’s register of dispensed medicines does not contain an exact dose in a structed form, we used Defined Daily Doses (DDD) to calculate the duration of the drug exposure. We assumed 0.9 DDDs for regularly used medicines based on calculations for regularly used medicines in an older adult population [24, 25]. Medicines were classified according to the anatomical therapeutic and chemical (ATC) system [26]. A constructed medication list was calculated for each individual in the cohorts; 31/32011 for cohort 1 and 31/122013 for cohort 2. From this constructed medication list, according to specified definitions, polypharmacy and use of PIM were identified.

We used indicator 1.1, ‘Medicines that should be avoided unless there are special reasons’ from the Swedish National Board of Health and Welfare report ‘Quality indicators for good drug therapy in elderly’ as the definition of PIM [13]. As the title states, it is medications that should be avoided in patients, 75 years and older, unless there are special reasons due to the higher risk of ADE. When prescribed it should be to a well-founded indication and the treatment should be evaluated at regular and frequent intervals. This definition of PIM was used in the national information campaign to follow-up the effects of the campaign and therefore it was used in this study also [13]. The following drug groups and substances are included in this definition of PIM: long-acting benzodiazepines, tramadol, propiomazine and medicines with anticholinergic effect. The use of PIM was identified from the constructed medication list by the medications ATC-codes.

Multimorbidity was defined as the number of chronic conditions. It was determined by using a validated assessment tool that captures chronic conditions grouped into 60 different categories of diagnoses [27]. All information about diagnoses for a two-year period prior to 31/3–2011 (cohort 1) and 31/12–2013 (cohort 2) were included.

### Data analyses

All variables were used as categories in the analyses. Gender was categorised as male or female and use of PIM; use or no use of PIM. Age was categorised into four groups: 75–79, 80–84, 85–89 and ≥ 90 and number of chronic conditions were divided into five groups or strata: none, one, two to four, five to seven and eight or more, chronic conditions. For the descriptive analysis of the cohorts, use of medications were divided into three strata; no-use of medications, use of 1 to 4 medications and use of five or more medications. A first descriptive analysis was performed of the two cohorts in the different strata of the variables age, gender, use of PIM, number of chronic conditions and number of medications. The differences were analysed using a chi-square test. A significance level (α) of 0.05 and 0.001 was used. Since polypharmacy is a known risk factor for ADEs, we wanted to analyse the prevalence of polypharmacy in the different strata [8]. Therefore, the number of medications was divided into two strata for the rest of the analyses; no use to use of four medications (< 5, no-polypharmacy) and use of five or more medications (≥5, polypharmacy).

We then described the cohorts from use of PIM in different strata of the variables age, gender, number of chronic conditions and polypharmacy and analysed the changes between the 2011 and 2013 cohorts. The cohorts were compared using a chi-square test.

The cohorts were then described and analysed from use of polypharmacy in different strata of the variables age, gender and number of chronic conditions. The cohorts were compared using a chi-square test. Significance levels (α) of 0.05 (*) and 0.001 (**) were used.

Logistic regression was then used to analyse how the different strata of the variables from 2011 were associated to the use of PIM 2013. Here only individuals present in both cohorts were included. We created four models; model A adjusted for PIM and age, model B adjusted for PIM, age and gender, model C adjusted for PIM, age, gender and number of chronic conditions, model D adjusted for PIM, age, gender, number of chronic conditions and polypharmacy. A logistic regression was performed to analyse how the different strata of the variables from 2011 were associated with decreased use of PIM 2013. We created four models: model A adjusted for age and gender; model B adjusted for age and gender and number of chronic conditions; model C adjusted for age, gender and polypharmacy; model D adjusted for age, gender, number of chronic conditions and polypharmacy. The purpose for this analysis was to investigate the variables association to have a decreased use of PIM between 2011 and 2013. The results for the logistic regressions are presented as odds ratio (OR) with 95% confidence interval (95% CI).

We used STATA version 14.0 (Stata Corporation, Texas, USA) for statistical analyses.

## Results

The number of individuals in the 2011 cohort was 15,361 and for 2013 it was 15,945 individuals. Of these, 11,973 (78%) individuals were present in both cohorts. The mean age in both cohorts was 82 years. However, the 2013 cohort had a higher prevalence of individuals 75–79 and 90+ compared to 2011. Prevalence of PIM decreased from 10.60 to 7.04% (*p*-value, < 0.001). The prevalence of chronic conditions increased over time. Five to seven chronic conditions increased from 20.55 to 23.66% and eight or more chronic conditions increased from 7.72 to 9.48% (*p*-value, < 0.001).

Non users of medications decreased from 20.82 to 19.19%, the use of 1–4 medications increased from 46.57 to 47.39% and the prevalence of polypharmacy from 32.62 to 33.41% (*p*-value < 0.001) (Table 1).

**Table 1:** Descriptive analysis of the two cohorts from 2011 and 2013

Variables		2011 (n)	%	2013 (n)	%	*p*-value
Total		15,361		15,945		
Age	75–79	6027	39.24	6472	40.59	
	80–84	4751	30.93	4733	29.68	
	85–89	3029	19.72	3021	18.95	
	≥90	1554	10.12	1719	10.78	< 0.05
Gender	Women	8907	57.98	9167	57.49	
	Men	6454	42.02	6778	42.51	0.377
Use of PIM	No	13,733	89.40	14,823	92.96	< 0.001
	Yes	1628	10.60	1122	7.04	< 0.001
Number of chronic conditions	0	2117	13.78	1762	11.05	
	1	2342	15.25	2076	13.02	
	2–4	6559	42.70	6822	42.78	
	5–7	3157	20.55	3773	23.66	
	≥8	1186	7.72	1512	9.48	< 0.001
	No-medications	3198	20.82	3060	19.19	
Polypharmacy	1–4	7153	46.57	7557	47.39	
	**≥5**	5010	32.62	5328	33.41	< 0.001

Use of PIM decreased in all strata of the variables. Among patients with chronic conditions, the greatest decrease was seen in two to four chronic conditions from 4.28 to 2.75% (*p*-value, < 0.001) (Table 2). Use of PIM decreased among patients with no-polypharmacy from 3.24 to 2.13% (*p*-value < 0.001) and polypharmacy from 7.36 to 4.91% (*p*-value < 0.001).

**Table 2:** Use of potentially inappropriate medication in 2011 and 2013

Variables	Categories	Use of PIM 2011		Use of PIM 2013		*p*-value
		No (%)	Yes (%)	No (%)	Yes %	
Age	75–79	5442 (35.43)	585 (3.81)	6049 (37.94)	423 (2.65)	< 0.001
	80–84	4278 (27.85)	473 (3.08)	4383 (27.49)	350 (2.20)	< 0.001
	85–89	2665 (17.35)	364 (2.37)	2815 (17.65)	206 (1.29)	< 0.001
	≥90	1348 (8.78)	206 (1.34)	1576 (9.88)	143 (0.90)	< 0.001
Gender	Women	7815 (50.88)	1092 (7.11)	8427 (52.85)	740 (4.64)	< 0.001
	Men	5918 (38.53)	536 (3.49)	6396 (40.11)	382 (2.40)	< 0.001
Number of chronic conditions	0	1970 (12.82)	147 (0.96)	1689 (10.59)	73 (0.46)	< 0.001
	1	2144 (13.96)	198 (1.29)	1982 (12.43)	94 (0.59)	< 0.001
	2–4	5901 (38.42)	658 (4.28)	6384 (40.04)	438 (2.75)	< 0.001
	5–7	2728 (17.76)	429 (2.79)	3433 (21.53)	340 (2.13)	< 0.001
	≥8	990 (6.44)	196 (1.28)	1335 (8.37)	177 (1.11)	< 0.001
Polypharmacy	< 5	9854 (64.15)	497 (3.24)	10,278 (64.46)	339 (2.13)	< 0.001
	≥5	3879 (25.25)	1131 (7.36)	4545 (28.50)	783 (4.91)	< 0.001

When analysing changes in prevalence of polypharmacy vs no-polypharmacy, the prevalence of polypharmacy increased in patients aged 80–84 years from 10.27 to 10.50% (*p*-value < 0.05) and males from 12.34 to 13.47% (*p*-value < 0.05) (Table 3). In patients with one chronic condition, the prevalence decreased from 2.68 to 1.99 (*p*-value < 0.05).

**Table 3:** Use of polypharmacy in 2011 and 2013

Variables	Categories	Number of medications 2011		Number of medications 2013		*p*-value
		< 5 (%)	≥5 (%)	< 5 (%)	≥5 (%)	
Age	75–79	4303 (28.01)	1724 (11.22)	4662 (29.24)	1810 (11.35)	0.429
	80–84	3173 (20.66)	1578 (10.27)	3058 (19.18)	1675 (10.50)	< 0.05^*^
	85–89	1925 (12.53)	1104 (7.19)	1850 (11.60)	1171 (7.34)	0.063
	≥90	950 (6.18)	604 (3.93)	1047 (6.57)	672 (4.21)	0.895
Gender	Women	5793 (37.71)	3114 (20.27)	5987 (37.55)	3180 (19.94)	0.702
	Men	4558 (29.67)	1896 (12.34)	4630 (29.04)	2148 (13.47)	< 0.05^*^
Number of chronic conditions	0	1846 (12.02)	271 (1.76)	1554 (9.75)	208 (1.30)	0.348
	1	1931 (12.57)	411 (2.68)	1759 (11.03)	317 (1.99)	< 0.05^*^
	2–4	4571 (29.76)	1988 (12.94)	4849 (30.41)	1973 (12.37)	0.079
	5–7	1592 (10.36)	1565 (10.19)	1941 (12.17)	1832 (11.49)	0.399
	≥8	411 (2.68)	775 (5.05)	514 (3.22)	998 (6.26)	0.720

Significant *P*-value < 0.05.

In the full model those having PIM 2011 had the highest odds of having PIM 2013 (OR 15.10 CI 95% 12.91–17.91) (Table 4). The number of chronic conditions was the only other variable that had significantly increased odds of having PIM 2013 in the full model. From two to four chronic conditions (OR 1.36 CI 95% 1.03–1.78) to eight and more (OR 1.80 CI 95% 1.25–2.58) the OR of having PIM 2013 increased slightly in each stratum of chronic conditions. Polypharmacy (OR 1.18 CI 95% 0.99–1.40) did, however, not increase the odds of having PIM compared to no-polypharmacy in the full model. When analysing the association of the variables from 2011, individuals with polypharmacy had the highest probability of deprescribing PIM (OR 4.64 CI 95% 3.96–5.44) in model C. The number of chronic conditions associated with deprescribing PIM were highest in individuals with 5 to 7 (OR 2.82 CI 95% 2.10–3.80) and 8 or more (OR 2.78 CI 95% 1.94–3.99) number of chronic conditions in model B. However, the effect was reduced in the full model (OR 1.42 CI 95% 1.04–1.94) (model D) while polypharmacy still had the highest probability to decreased use of PIM (OR 4.40 CI 95% 3.72–5.22) (Table 5).

**Table 4:** Odds ratios (OR) to have PIM in 2013 in nested models for patients in 2011 (*n* = 11,973)

Variables in 2011	Categories	Model A	Model B	Model C	Model D
		OR (CI 95%)	OR (CI 95%)	OR (CI 95%)	OR (CI 95%)
PIM	No	1	1	1	1
	Yes	16.64 (14.28–19.40)*	16.70 (14.31–19.47)*	16.01 (13.71–18.70)*	15.10 (12.91–17.91)*
Gender	Women	1	1	1	1
	Men	0.91 (0.78–1.06)	0.90 (0.77–1.06)	0.90 (0.77–1.06)	0.91 (0.77–1.07)
Age	75–79		1	1	1
	80–84		0.97 (0.82–1.16)	0.96 (0.81–1.15)	0.96 (0.80–1.15)
	85–89		0.84 (0.68–1.05)	0.83 (0.66–1.03)	0.82 (0.66–1.03)
	≥90		1.01 (0.75–1.36)	1.01 (0.75–1.36)	1.00 (0.74–1.35)
Number of chronic conditions	0			1	1
	1			1.24 (0.90–1.70)	1.23 (0.89–1.69)
	2–4			1.40 (1.07–1.83)*	1.36 (1.03–1.78)*
	5–7			1.52 (1.13–2.04)*	1.43 (1.06–1.93)*
	≥8			1.96 (1.38–2.78)*	1.80 (1.25–2.58)*
Polypharmacy	< 5				1
	≥5				1.18 (0.99–1.40)

* Significance

**Table 5:** Odds ratios to deprescribe PIM in 2013 for patients in 2011 (*n* = 11, 973)

Variables in 2011	Categories	Model A	Model B	Model C	Model D
		OR (CI 95%)	OR (CI 95%)	OR (CI 95%)	OR (CI 95%)
Gender	Women	1	1	1	1
	Men	0.66 (0.56–0.78)*	0.66 (0.56–0.77)*	0.71 (0.61–0.84)*	0.71 (0.60–0.83)*
Age	75–79	1	1	1	1
	80–84	1.07 (0.89–1.28)	1.03 (0.86–1.23)	1.00 (0.83–1.19)	0.99 (0.83–1.19)
	85–89	1.26 (1.03–1.55)*	1.19 (0.97–1.47)	1.14 (0.93–1.41)	1.16 (0.87–1.56)
	≥90	1.62 (1.23–2.14)*	1.57 (1.19–2.08)*	1.39 (1.05–1.85)*	1.39 (1.05–1.85)*
Number of chronic conditions	0		1		1
	1		1.29 (0.92–1.81)		1.14 (0.81–1.62)
	2–4		1.68 (1.26–2.24)*		1.16 (0.87–1.56)
	5–7		2.82 (2.10–3.80)*		1.42 (1.04–1.94)*
	≥8		2.78 (1.94–3.99)*		1.11 (0.76–1.62)
Polypharmacy	< 5			1	1
	≥5			4.64 (3.96–5.44)*	4.40 (3.72–5.22)*

*Significance

Model A adjusted for PIM and gender; Model B adjusted for PIM, age and gender; Model C adjusted for PIM, age, gender and number of chronic conditions; Model D adjusted for PIM, age, gender, number of chronic conditions and polypharmacy.

Model A adjusted for age; and gender Model B adjusted for age and gender and number of chronic conditions; Model C adjusted for age, gender and polypharmacy; Model D adjusted for age, gender, number of chronic conditions and polypharmacy.

## Discussion

Use of PIM decreased in all the variables, age, gender, number of chronic conditions and polypharmacy during a national information campaign to reduce PIM. However, the decrease was more evident in women, patients with polypharmacy and patients with two to four chronic conditions. The group that had the highest probability to deprescribe PIM during the study period was patients with polypharmacy and high number of chronic conditions.

The positive trend of the reduced prevalence of PIM users found in this study corresponds with results from other reports in Sweden during the same time period [28–30].

In 2005 the prevalence of PIM was found to be 17% in a Swedish older adult population and a national comparison showed that use of PIM had decreased by 44% between 2005 to 2014 [31, 32]. Use of PIM and polypharmacy is associated with increased risk for ADEs and hospitalisation [9, 33].

In our study, the prevalence of polypharmacy stayed relatively stable, but number of chronic conditions increased. The fact that polypharmacy did not increase significantly while the number of chronic conditions increased is an interesting finding. One could think that if multi-morbidity is increasing that polypharmacy would follow. However, the use of medications did increase, just not polypharmacy in comparison with the rest of the population.

The decrease in PIM in this study was not paralleled by a decrease in polypharmacy. The most common drug classes in patients 75 years and older with polypharmacy are not PIM (according to our definition) but cardiovascular drugs (including antithrombotic agents), analgesics and psychotropic drugs [34]. These are also the most commonly used drugs in adverse drug events, such as bleeding or bruising, which are associated with antithrombotic agents, or dizziness and unsteadiness due to psychotropic medicines [4].

It can be stated that based on this single quality indicator, the use of PIM has improved and thereby the quality of medication treatment in older adults. However, it does not affect the total quality of medications use. The information campaign was a success as regards that it reduced the use of PIM, especially in patients with high number of chronic conditions and polypharmacy. However, it did not reduce polypharmacy, which is also an important factor for quality in medication use in older adults [20, 35–37].

The results in this study show that the use of a clear and simple quality indicator as decreased use of PIM can improve the quality of medication treatment in older adults. However, to affect other factors of importance for the quality of medication treatment, a combination of quality indicators may be better to use. For example, the STOPP criteria, a collection of quality indicators, reduced the number of ADEs when implemented in a hospital setting in a study from Cork University Hospital [38]. The complete collection of quality criteria in “Quality indicators for good drug therapy in elderly” from the Swedish National Board of Health and Welfare can be used in the same way [13]. The effect is more complex to evaluate on a population level, but the clinical effect in the individual is greater.

### Strengths and limitations

The definition of PIM used in this study is stricter in its definition and includes fewer drugs and drug classes than other definitions [13, 15]. For example, we do not include nonsteroidal anti-inflammatory drugs (NSAID) or cardiovascular drugs except for disopyramide. However, our definition from the Swedish National Board of health and Welfare is commonly used in Sweden as an indicator for quality of drug treatment in older adults, both nationally and by county councils and is therefore relevant in this setting. On the other hand, this means that our results cannot be directly translated to other settings where the definition of PIM is broader.

The county council’s register, used in this study to identify use of medication, includes medications that are prescribed, and pharmacy dispensed for all inhabitants in Blekinge. Use of illegal drugs or over the counter drugs are not included in this study. In 2011, the Medical Products Agency in Sweden presented a study that indicates that 11% of the Swedish population had at some time bought prescription drugs from non-approved pharmacies [39]. The method to construct a medicine list on dispensed prescribed drugs from the inclusion date for the cohorts and three months back, allowed us to determine, as closely as possible to index date, as to what the patient was using. The limitation of this method is the possibility of underestimating the use of medications prescribed to be used as needed. This because they are dispensed more rarely than every three months. The patient’s compliance, i.e. if they were using their medications as prescribes, were we not able to take into consideration when determining use of PIM. However, the method used, to identify use of medication in this study, is validated and the time period of three months has been found to be the most optimal [25]. Factoring in the Swedish system that has a high cost threshold, there is a limited risk of hoarding medications [23].

Number of chronic conditions, that are used in this study to measure multimorbidity, is dependent on the quality of registration of diagnoses in the medical records [27]. The recording of diagnoses in this study has not been validated. However, we used registered diagnoses from a two-year period from both primary- and secondary care to get as close to total coverage as possible. There are other multimorbidity estimates that are constructed by giving different diagnoses a weight as to how much the diagnosis contributed to need of care or cost [40, 41]. In our definition, all chronic conditions contribute equally to the estimate, i.e. an expression of the complexity of a patient’s morbidity and their need of care.

We used two different cohorts in this study instead of following one cohort over time when analysing prevalence of PIM and polypharmacy in relation to studied variables. If we had used only the individuals present in both cohorts (78%) the results would have been affected by the fact the population had aged.

Blekinge County is a small county in Sweden, both in terms of population and area, and has a relatively simple organisation of health care service, which makes it easy to include data from primary care centres, both public and private, and from secondary care. Our results are applicable to populations with older adults in similar settings.

## Conclusion

Our results show that the use of PIM in older adults decreased in all strata of number of chronic conditions and in patients with polypharmacy. The results also show that the complexity of older adult patient’s use of medications is increasing. The older adult population is growing together with the number of chronic conditions. However, while the use of medications in the older adult population increased, the prevalence of polypharmacy remained stable.

With clear and simple quality indicators it is possible to improve quality of drug treatment in the older adult population during a national information campaign to reduce PIM. The challenge is to create and evaluate indicators that measure quality of drug treatment in a population that has clinical value in an individual patient. More focus and effort needs to be directed to methods for optimisation of drug treatment in the individual. Quality indicators for evaluating drug treatment in a population need to continue to be developed and implemented. Future studies need to focus on methods for optimising and evaluating the quality of drug treatment when including multi-morbidity and polypharmacy in the context.

## Data Availability

The datasets generated and/or analysed during the current study are not publicly available due to individual privacy being compromised. Due to ethical restrictions and the sensitive nature of the data, data are only available after ethical approval according to Swedish regulation: http://www.epn.se.

## Abbreviations

ADE: Adverse Drug Events
ATC: Anatomical Therapeutic and Chemical
CI: Confidence Interval
DDD: Defined daily doses
OR: Odds ratio
PIM: Potentially Inappropriate Medications
STOPP: Screening Tool of Older Persons’ potentially inappropriate Prescriptions
